# “Tree to fight hunger”: determinant of *enset* market participation and intensity of participation: the case of Southwest Ethiopia

**DOI:** 10.1016/j.heliyon.2022.e08721

**Published:** 2022-01-12

**Authors:** Engida Gebre, Yaregal Tilahun, Benyam Tadesse, Kusse Haile, Tewdros Legesse

**Affiliations:** a Department of Agricultural Economics, Mizan Tepi University, Ethiopia; b Department of Plant Sciences, Mizan Tepi University, Ethiopia

**Keywords:** Market participation, Enset, Marketed surplus, Heckman two-step, Southwest Ethiopia

## Abstract

Kaffa, Sheka, and Bench sheko Zone in the Southwest region of Ethiopia are known for enset farming. The objective of this study was to identify factors constraining market participation of enset producers and marketed surplus. Data were obtained from a sample of 657 enset producers. Heckman's two-stage model was used to identify the determinants of enset products market participation and marketed surplus. Heckman's two-stage selection model results showed that family size, level of education, farming experience, land allocation, livestock ownership, and access to training had significantly influenced market participation decision while family size, level of education, farming experience, livestock ownership, access to transport, quantity enset produced, off-farm income and inverse Mill's ratio (LAMBDA) influenced significantly the extent of marketed surplus. Based on the findings of the study, we suggest that the government and concerned stakeholders should focus on promoting improved enset variety, encouraging the use of labor-saving technology, strengthening the existing social services, promoting farmers' cooperatives, empowering women, improving market linkage, and competitive market should be created.

## 1 Introduction

*Enset***(***Enseteventricosum* (Welw.) Cheesman**)** is a perennial herbaceous, monocarpic, and monocotyledonous crop th*at* belongs to the order *Scistaminae* and family *Musaceae* (Shigeta, 1991)*. Enset* been grown in Ethiopia for more than 10,000 years ago (Birmeta et al. 2004; Pankhurst, 1996; Shigeta, 1990); grows in the mid-altitudes to the highlands (about 1500–3000 m) in south, southwest, and central regions (Bezuneh and Feleke, 1966; Pijls et al., 1995; Westphal et al., 1975). Where domesticated, enset grows at altitudes ranging from 1,200 to 3,100 masl, but grows best at 2000–2750 m.a.sl (Brandt et al., 1997).

Enset is a multipurpose crop where all portions of the plant are used for different purposes and it serves as a staple and/or co-staple food for more than 20 million people that inhabit in the south and Southwestern Ethiopia (Brandt et al., 1997; Pijls et al., 1995; Negash and Niehof, 2004; Woldesenbet, 2013; Yemataw et al., 2014, Borrell et al., 2019; Haile et al., 2020 and Mulatu, 2021). These areas are among Ethiopia's most densely populated, with more than 11 ethnic groups living there, each with its own culture and agricultural methods (Tsegaye and Westphal, 2002). Kocho, bulla, and amicho are the most common foods obtained from Enset (Ayele and Sahu 2014; Nuri and Jema, 2016; Tessema et al., 2017; Haile et al., 2020; Mulatu, 2021). Enset benefits the surrounding ecosystem by improving soil nutrient balance (Elias et al., 1998), providing shade and therefore cooling the environment, and being a part of farming systems with high biodiversity (Bizuayehu, 2008; Zerfu et al., 2018).

Enset is thought to be relatively high drought tolerant (Garedew et al., 2017; Zerfu et al., 2018). So it contributes to food security for millions of Ethiopia's population (Ayele and Sahu 2014; Yemataw et al., 2016), and survive torrential rain, flooding, and frost damage (Degu and Workayehu, 1990). The country can generate more from the plant to become self-sufficient in food, and it may serve as one of the greatest food ingredients to meet everyone's daily nutritional needs. It is an important economic and socio-cultural crop for a large number of smallholder households throughout the country, and it is also utilized as a traditional medicine (Olango et al., 2014). As per the central statistical agency report of (CSA, 2016), Ethiopia harvested 130,630,473 enset (warqe) plants, yielding 34,723.6 tonnes of Kocho, 12,259.4 tonnes of Bulla, and 311.3 tonnes of *amicho* during the production season.

Despite its huge potential, *enset* production has not been fully exploited and promoted in the country. Sever*al* factors, such as poor marketing infrastructure, use of traditional technologies, limited supply, and lack of marketing support services and market information, and limited credit services have contributed to under exploitation of *enset* production potential (Spring et al., 1997; Hailu, 2016; Mulatu, 2021).

The southwest is one of the highest green areas considered as the amazon of Ethiopia, having fertile land where different plant species and the crop can grow within it. Due to this fact, the livelihood of the majority of people in the south and southwest part depends on root and tuber crops. They have a great contribution to income generation, assuring food security, provision of food energy, and resource base conservation (Gebremedhin et al., 2001; Meyer, 2011). Hence, *enset* is one of the innate food security root and tuber crops (Adimassu et al., 2014) which serve as home consumption and marketing as the main food crop in the place where the study was conducted.

A market-oriented production system is a strategy to promote stimulation of consumption and increase production by seeking extra output (Haji, 2008; Schulte-Geldermann, 2013; Tufa et al., 2014). However, the output of *enset* is not providing the expected amount of benefit for the households in the three zones due to different constraints such as reluctance to adopt improved clones in terms of yield and disease resistance (Shumbulo et al., 2012), lack of initiation from different stakeholders to develop new processing technology (Valentina, 2014) to adopt it from other areas of the country, post-harvest losses (Chaka 2016), poor infrastructural since the study area is politically marginalized areas of the country, the existence of inefficient *enset* marketing characterized by high margins and poor marketing facilities and services, improper or traditional agronomic practice are the major challenge in *enset* production (Spring et al., 1997; Shumbulo et al., 2012; Tuffa et al., 2017). Besides, the role of enset is poorly understood across many geographic regions and production systems (Frison et al., 1997) particularly in the study area and marketing of the product is subjected to a seasonal variation where surplus supply at the harvest time is the main feature (Mohammed and Tariku, 2010; Tamire and Argaw, 2015; Valentina, 2014; Nuri and Jema, 2016; Yemataw et al., 2017).

In addition to the above challenges, the benefit accrued from producing *enset* for marketing purposes is not well known by producing households and till not identified. Then, this, in turn, results in low market participation of *enset* producing households. In addition, to the best of researchers’ knowledge, no studies were found that provide empirical study has been done on improving the marketing of *enset* products in the area except research conducted by (Mulatu, 2021; Garedew et al., 2017). Therefore, investigation of *enset* products market participation of *enset* producer is essential to provide information on the potential constraints that need to be alleviated and opportunities that need to be utilized (Valentina, 2014; Abebe and Paul, 2015). Therefore, the current study focuses on the socio-economic, institutional, and political factors which constrain the *enset* product market participation.

## 2 Research methodology

### 2.1 Description of the study area

The study was conducted in Kaffa, sheka*,* and Bench-Sheko zones of southwest Ethiopia. Bench-sheko zone is found at a distance of about 561 km from Addis Ababa and 830 km from the regional capital. Agro-ecologically, the zone is found at an altitude range from 500 to 3,000 masl. The zone is found at 34^o^45′-36^o^10′ east and 5^o^40′-7^o^40′ north. The temperature of this area ranges from 15.1 °C to 27.5 °C, while the annual rainfall ranges from 400 to 2,000 mm (Bench sheko zone, 2019).

Kaffa zone is found at a distance of about 460 km from Addis Ababa and 690 km from the regional capital. The zone is found at the latitude of **7°**10′46.78**″** and longitude of 36°2′52.44". The estimated terrain elevation above sea level is 1795 m. The annual temperature ranges from 14.1 °C to 26.5 °C, while the annual rainfall ranges from 400 to 2,000 mm.

Sheka Zone is located at 7^O^24′-7^O^52′ north latitude and 35^O^13′-35^O^35′ east longitude, at a distance of 700 km from Addis Ababa. It covers about 2175.25 km^2^, out of which, 47% is covered by forest, including bamboo. The altitude is between 900-2700 m.a.s.l. and it receives a lot of rain regularly (annual average) approximatley 1800–2200 mm and the average mean temperature ranges from 15.1 to 27.5 ^O^C. The rain-fed production system is most dominant and practiced by the majority of the farmers (Tadesse et al., 2021).

### 2.2 Data types, sources, and method of data collection

Both quantitative and qualitative data were used. To generate the data, both primary and secondary data sources were used. To collect primary data from enset producing farmers, a semi-structured questionnaire was prepared. The questionnaire was pre-tested and amended based on the feedback received during the pre-test. To reduce the difficulty of data collection, the enumerators who can speak the local language and are familiar with the culture were chosen and were trained on data collecting procedures. In addition to the questionnaire, focus group discussions and key informant interviews were conducted to seek additional information and/or cross-check the data. Moreover, the primary data results were supported by relevant secondary data sources like reports of journals, books, Central Statistics Agency (CSA), zonal and district reports, among others.

### 2.3 Sampling procedure and sample size determination

#### 2.3.1 Enset producers

The target population for this study was smallholder *enset* producing farm households. To select a representative sample, a combination of purposive and three-stage sampling techniques were employed to select districts, *enset* crop-producing *kebeles,* and sample farm households. From the three zones, three major *enset* producing and marketing districts namely Chena, Masha, and Sheybench was purposely selected since they are potential producers of *enset*. Then, kebeles in the district were stratified based on the production levels. In the next stage from the selected districts, a total of 19 kebeles were selected randomly from the strata. Finally, from a total of (57,411) *enset* producing farm households in the three districts, 657 sample *enset* producing farmers were selected randomly based on probability proportional to the population size of the selected kebeles from each of strata by using the Kothar formula as indicated in Eq. (1).

(1) $$n=\frac{Z^{2}pqN}{\left(N-1\right)e^{2}+Z^{2}pq}\;$$

Where, n = sample size, Z^2^ = 95% = 1.96, e = level of precision (5%), p = the population proportion (assumed to be 0.5 for it provides the maximum sample size) and q = (1-p). Accordingly, the proportion of the required sample size from each selected district to represent the true population was described in (Table 1).

**Table 1:** Sample size determination of enset producers in selected districts.

Name of the District	Number of *enset* producer	Sample size
Sheybench	15092	173
Masha	241091	276
Chena	18228	208

Source: From Zonal Agricultural office (2018/19).

### 2.4 Methods of data analysis

Descriptive statistics and econometric models were used for analyzing the data collected from enset producing households.

Descriptive statistics such as mean standard deviation, percentage, and frequency; and descriptive tests like t-tests and chi-square were used.

### 2.5 Econometric analysis

Heckman sample selection (two-step) was used in this research to assess the association between depedent and independent variables. Heckman has developed a two-step estimation procedure model that corrects for sample selectivity bias. If two decisions are involved, such as participation and quantity of enset products sales, Heckman's (1979) two-step estimate approach is appropriate. The first stage of the Heckman model (“participation equation”) aims to capture factors affecting market participation decisions. This equation is used to create the “Inverse Mills Ratio,” a selectivity term that is added to the second stage “outcome equation” that explains factors affecting the quantity of *enset* products supplied to the market. Generally, the models help us to identify and evaluate the factors that influence smallholder farmers' decision to participate in the enset market, as well as the level of market participation. Specification of the Heckman two-step procedure, which is written in terms of the probability of enset product producers market participation ($Y_{1i}$) which is a discrete choice as indicated in Eq. (2).

(2) $$Y_{1i}=1\;if\;Y_{1i}^{*}\;>0\;\text{and ​}Y_{1i}=Y_{1i}^{*}\;\leq 0\;$$

(3) $$Y_{1i}^{*}=X_{1i}\beta_{1i}+\varepsilon_{i}\;i=1,2,3,4......n$$

Where $\;Y_{1i}$ is the probability of *enset* product producers' market participation; which is a dummy variable assuming the value of 1 for market participants and 0 otherwise. $Y_{1i}^{*}$ is a latent variable; $X_{1i}$ are the variables determining participation in the probit model; $\;\beta_{1i}$ are unknown parameters to be estimated in the probit regression model; $\varepsilon_{i}$ is a random error term as shown in Eq. (3). Then the factors can be reliably expected by truncated regression across n observations reporting values for $\;Y_{2i}$ by including an estimate of the inverse Mills ratios indicating ${\text{λ}}_{i}$ as an additional variable from the selection equation as indicated in Eqs. (4) and (5). The observation equation is more exactly written as follows:

(4) $$Y_{2i}=X_{2i}\beta_{2i}+\mu_{i}\lambda_{i}\;+\varepsilon_{i}$$

Where $\;Y_{2i}\;$ is the amount of marketed surplus in the second step; $X_{2i\;}$ are the independent variables determining the intensity of market participation; $\beta_{2i}$ are unknown parameters that show estimated in the market participation; $\mu_{i\;}$ is a parameter that shows the impact of selectivity bias on general role; $\varepsilon_{i}$ is the error term.

(5) $$\lambda_{i}=\frac{f\left(X_{i}\beta_{i}\right)}{1-f\left(X_{1i}\beta_{1i}\right)}$$

*f* ($X_{i}\beta_{i}$) is density function and 1-f (*X* i β*i*) is distribution function. The explanatory variables used in the model were described in (Table 2).

**Table 2:** Summary Hypothesize definition of dependent and independent variables.

Variables	Type	Description	Expected sign
Dependent Variables			
Market participation decision	Dummy	A binary variable indicating who participate in the market and it takes the value of 1 otherwise, 0	
The volume of *enset* product marketed	Continuous	The total amount of *enset* product marketed in kg	
Independent Variables			
Years of experiences	Continuous	Years of experience in several years	**+**
Sex of household	Dummy	1 if the household head is male and 0 otherwise	**+/-**
Education level	Continuous	Level of education completed in years of the household head	**+**
Household size	Continuous	Number of people in the household	
Livestock ownership	Continuous	The number of livestock owned by the household	**+/-**
Land under *enset*	Continuous	Total land size of *enset* owned by the household	**+**
Extension ​contact frequency	Continuous	Frequency of the extension visit of the farm households	**+**
Credit utilization	Dummy	1 if the household has access to credit; otherwise.	**+**
Market inform Access	Dummy	1 if a farmer has market information and 0 otherwise.	**+**
Access to training	Dummy	“1” for having access and “0” otherwise	**+**
Perception of price	Dummy	1 if relatively attractive and 0 if otherwise	**+/−**
Market distance	Continuous	Distance from the household's residence to the nearest market.	**-**
Quantity produced Non/off-farm income	Continuous Continuous	Quantity produced (kg) ETB	**+** **−/+**


Source: own assumption, (2019/20).

## 3 Results and discussion

### 3.1 Socio-demographic characteristics of *enset* producers

Descriptive statistics were used to describe the socio-economic and institutional characteristics of the households considered in the study of value chain analysis of *enset*. In the study, we explored survey data using descriptive statistical tools such as mean, frequencies, standard deviation, and percentages to give general descriptions about household characteristics. Moreover, the t-test and chi-square tests were used to measure the significance levels of the results. In this study, participant refers to farmers who produce *enset* and sell product to the market and those farmers who didn't sell *enset* product are considered as non-participants. The descriptive and inferential statistics results presented in Tables 3 and 4 shows that there was a statistically significant difference between participant and non-participant in terms of credit access, training, road accessibility, education level, land under *enset*, marketed surplus, and quantity produced.

**Table 3:** Descriptive statistics of Dummy Variables.

Variables	Percentage of participation category			
	Participants (429)	Non-participants (228)	χ2- value	p-value
Sex of household head				
Male	67	45	1.7863	0.181
Female	362	183		
Credit access				
Yes	190	72	10.0313∗∗∗	0.002
No	239	156		
Access to training				
yes	276	96	29.9547∗∗∗	0.00
No	153	132		
Perception of price				
Low	133	73	0.0834	0.959
Moderate	199	105		
High	97	50		
Road accessibility				
Yes	225	103	3.1493∗	0.076
No	204	125		
Mobile ownership				
Yes	208	102	0.8392	0.360
No	226	126		

Source: own survey (2019/20), ∗, ∗∗ and ∗∗∗ indicates 10%, 5% and 1% of significance probability level respectively.

**Table 4:** Descriptive statistics test for continuous independent variables.

Continuous variables	Participants	Non-participants		
	Mean (SD)	Mean (SD)	t-test	P-value
Household size	6.184 (2.321)	6.245 (2.433)	0.7508	0.3177
Education level	4.090 (3.709)	1.583 (2.948)	8.830 ∗∗∗	0.00
Years of experience	17.372 (8.528)	15.25 (8.227)	0.423	0.672
Land under *enset*	0.293 (0.201)	0.245 (0.158)	3.087∗∗∗	0.002
Livestock ownership	3.978 (1.909)	4.185 (2.001)	-1.304	0.192
Marketed surplus	160.056 (109.965)	103.723 (57.497)	17.383∗∗∗	0.00
Off/non-farm income (log)	4.011 (2.057)	3.856 (1.611)	0.470	0.6383
Quantity produced Market distance	362.699 (205.962) 8.937 (6.355)	202.995 (161.861) 8.304 (5.826)	10.158∗∗∗ 1.248	0.000 0.212

Source: own survey (2019/20), ∗, ∗∗ and ∗∗∗ indicates 10%, 5% and 1% of significance probability level.

This section begins by discussing the demographic characteristics of sample respondents on different variables. A combination of different descriptive analyses (means and standard deviation), inferential statistics (t-test and *X*^2^-test) and statistics for explanatory variables of sample households were performed on the household level data to inform the subsequent empirical data analysis. The descriptive and inferential results presented in Tables 3 and 4 shows that there was a statistically significant difference between participants and non-participants in terms of credit access, training, road accessibility, educational level, land under *enset*, marketed surplus and quantity produced.

As shown in Table 3, out of the total sample respondents, 429 (65.30%) were participants in *enset* product market whereas 228 (34.70%) households are non-participants. As it was proposed previously, the sex of households was a dummy variable and it was categorized as female and male. Therefore, from the total sample households, 545 were female and 112 were male. Therefore, from the total participant households, 84.38 % and 15.62 % sample households are female and male-headed households respectively. While 80.26 % and 19.74 % of the household are female and male non-participant households respectively. The result is in line with the finding of Tesema et al. (2017) and Mulatu (2021) who found that the participation of female-headed households for enset production was higher than that of male-headed households.

The mean years of experience for participants were 17.372 years and the mean years of farming experiences for non-participants was 15.25 years. The mean education level of participant households was 4.090 years schooling whereas the mean educational level for non-participants households was 1.583 years of schooling and there was a statistically significant mean difference between the two groups at a 1% level of significance. This result is in line with Tessema et al. (2017) and Haile et al. (2020) study who stated that the educational level of participants was higher than that of non-participants.

The mean livestock ownership of participant households was 3.978 and 4.185 for the non-participants respectively. The mean household size of participant households was 6.184 persons and 6.245 for non-participants. The average distance taken for the participant household to travel from the residence to the nearest marketplace was 8.937 km and 8.304 km for non-participants which were a statistically significant mean difference between the two groups at a 1% level of significance. The finding was consistent with the study of Tessema et al. (2017), Mulatu (2021), and Haile et al. (2020) which indicated that the distance of *enset* participant households was lower than that of non-participants. The households can earn additional income by engaging in various off-farm activities. This is believed to raise their financial position to acquire new inputs. The mean off/non-farm income for participants' households was birr log 4.011 whereas for non-participants birr log 3.856 respectively.

The mean land allocated for *enset* was 0.293 ha for participants' households whereas; the non-participants’ mean cultivable land was 0.245 ha and this was significant at a 1% level. This size is very few concerning the national average households' land size of 1.37 ha (CSA, 2014). This result is in line with the research conducted by Haile et al. (2020) and Mulatu (2021) which revealed that there is a statistical difference in enset land allocation among participants and non-participants. The mean quantity produced of *enset* product was 362.699 kg/ha for participants’ households and the mean quantity produced for non-participants was 202.995 kg/ha. This finding is also similar to the research finding by Haile et al. (2020) which found that households with a higher value of production sold their produce with better market participation.

### 3.2 Determinants of *enset* product market participation and intensity of participation

Market participation is defined as the quantity or proportion of harvested output that is marketed. Hence, households' market participation was expressed through the sale of *enset* at different levels. Double hurdle, Tobit and Heckman models could be used to estimate the effect of hypothesized variables on market participation and level of sales. Hence, much emphasis had been given to identifying the relatively better econometric model that best captures the objective of the study.

Heckman's two-stage selection econometric model was adopted because the estimation result of market participation and intensity of participants suggested that there is sample selectivity bias since the IMR is statistically significant at a 5% significance level as indicated in appendix Table 4. The result of Heckman maximum likelihood model (ML) outputs also indicated that the two equations are interdependent because the null hypothesis revealed that the market participation and level of participation are independent are rejected at a 5% significance level. Consequently, the two equations are estimated simultaneously using the Heckman selection model.

#### 3.2.1 Determinants of *enset* product market participation

Results of first-stage probit model estimation of the determinants of *enset* market participation decision of the sampled households are given in Table 5. The overall goodness of fit of the probit model is statistically significant at less than 1% probability level. The Hausman specification test result in the appendix table revealed that the model was a good fit. The overall model is significant at 0.0000 levels as indicated by the log pseudo-likelihood value of -319.581. The model has correctly predicted 72.34% of the observations, with a significant chi-square value of 206.59. A total of fourteen potential explanatory variables (six dummy and nine continuous) were selected and entered into the selection/probit model. Out of the fourteen explanatory variables, six of them were found to determine the probability of participating in *enset* market significantly. These are household size, education level, experience, land allotted for *enset*, livestock ownership, and access to training.

**Table 5:** Maximum likelihood estimates first-stage probit estimation (Marginal effects after probit).

Variables	Coef.	dy/dx	Std.Err.	Z	P > Z
Sex of household	.0101	-.079	.0326	0.31	0.756
Household size	.016∗∗	.046	.007	2.21	0.027
Education level	.013∗∗∗	.052	.005	2.68	0.007
Years of experience	.007∗∗∗	.017	.002	2.94	0.003
*Enset* land allocation	.130∗∗	.443	.056	2.33	0.020
Livestock ownership	-.036∗∗∗	-.110	.013	-2.74	0.006
Credit access	-.029	.070	.020	-1.46	0.144
Frequency of extension contact	.007	.016	.009	0.79	0.432
Access to raining	.097∗∗∗∗	.099	.021	4.50	0.000
Perception on price	-.006	-.007	.011	-0.51	0.608
Market information	-.031	-.057	.021	-1.47	0.141
Access to transport	-.009	-.001	.019	-0.49	0.626
Quantity produced	.0011	.0008	.0011	0.11	0.911
Non/off farm income (log)	-.003	-.0199	.0027	-1.10	0.272
_cons	.693		.1042	6.66	0.000

∗∗∗, ∗∗, and ∗ indicate statistical significance at 1%, 5%, and 10%, respectively. Std. Err = Standard Error; Source: Own survey results, 2018/19.

Household size influences *enset* product market participation decision significantly and positively at less than 1% significance level. In contrast to the prior expectation, household size has a negative relationship with the *enset* market participation. The marginal effect of the variable also confirms that a one-person increase in the household of *enset* producer households leads to an increase in the probability of participation in *enset* market by 4.6%. This may be explained by the fact that farmers who have a large number of households sizes tend to involve in different activities during *enset* production time. The result was in contrast with the finding of Woldesenbet (2013), Hailu (2016), Tessema et al. (2017) and Mulatu (2021) who found that *enset* was mainly produced for home consumption and those households with higher family size supplied lower surplus amount and earned lower gross income.

The educational level of the household head was affected market participation positively and significant at less than 1% level of significance. A one-year increase in education level increases the probability of household's market participation by 5.2 %, keeping other things remain constant. Education was believed to give individuals fundamentals that help them to gather information, interpret the information, make good production, and supply decisions to the market (Haile et al., 2020). This is because being literate may support them to receive and comprehend information on production and marketing of *enset* products better. In line with this, Eshetu (2016) and Haile et al. (2020) revealed that household heads who attended formal education have good information and can participate in enset market. Livestock holding measured in tropical livestock unit (TLU) is found to negatively and significantly influence the probability of market participation decision. The result revealed that a unit increase in livestock ownership in TLU decreases the probability of household's market participation by 11.1%, keeping the other things constant. This is because having more livestock creates a better opportunity for a diversified source of farm income. This is in line with the fining of Tessema et al. (2017) and Mulatu (2021) who found that households who have more livestock showed lower participation in selling *enset* products but in contrast with the finding of Nuri and Jema (2016) who showed a positive relation between livestock ownership with *enset* production and marketing.

Another socio-economic characteristic that affects households' market participation decisions is the size of land allocated for enset production. The size of land allocated for *enset* production has significantly affected the decision of market participants at a 5% significance level. The average partial effect of this variable implies that for a hectare increase in land allocated for wheat, the probability of market participation increases by 13% which in turn leads to the increased probability of deciding to participate in the product's market by the households. The findings of Tessema et al. (2017) confirmed this finding by indicating a positive relationship between variables. Access to training significantly and positively influences *enset* market participation. The result showed that those households who had access to training increases the probability of households participating in the *enset* market by 9.9%, all other factors held constant. Farmers who have taken training would be aware of the quality of *enset* producing to be supplied to the market. So, giving training and awareness to enset producer households at right time with the right place is crucial to increase their skill and knowledge and can increase enset market participation.

#### 3.2.2 Determinants of *enset* marketed surplus

Result Heckman second stage shows that the null hypothesis for the test is that all coefficients are jointly zero. The overall goodness of fit for the Heckman selection model is statistically significant at a probability of less than 1%, according to model chi-square tests using appropriate degrees of freedom. This demonstrates that the independent factors included in the selection model regression together explained the degree of marketed surplus. Seven explanatory variables namely, household size, education level, years of experience, livestock ownership, quantity produced, off/non-farm income (log), and mills lambda significantly affected marketed surplus level. According to the model output, Lambda (IMR) or selectivity bias correction factor has a positive impact on farm households’ *enset* product market participation at a 5% significance level. And, the positive sign of the IMR shows that the existence of unobserved factors that positively influence both participation decision and level of *enset* output marketed. Moreover, rho is positive, indicating that unobservable factors are positively correlated with one another (Table 6).

**Table 6:** Maximum likelihood estimates of second-stage Heckman selection estimation of determinants of market participation intensity.

Variables	Truncated regression			
	Coefficient	Std.Er.	Z	P > Z
Sex of household	-.148	.200	-0.74	0.458
Household size	.1256∗∗∗	.044	2.82	0.005
Education level	.121∗∗∗	.020	6.05	0.000
Years of experience	.043∗∗∗	.011	3.82	0.000
*Enset* land	.259	.423	0.61	0.540
Livestock ownership	-.262∗∗∗	.062	-4.21	0.000
Credit access	.234	.155	1.51	0.130
Frequency of extension contact	.089	.057	1.55	0.121
Access to raining	.131	.143	0.91	0.361
Perception on price	.017	.079	0.22	0.823
Market information	-.231	.135	-1.71	0.087
Market distance	.018	.011	1.56	0.118
Access to transport	-.115	.133	-0.87	0.387
Quantity produced	.002∗∗∗	.0003	7.87	0.000
Off/non-farm income (log)	-.038∗∗	.0189	-2.01	0.045
_cons	-1.403∗∗∗	.4004	-3.50	0.000
Mills lambda	.155∗∗	.0763	2.04	0.042
Rho	0.839			
Sigma	.185			

Source: own survey (2019), ∗, ∗∗ and ∗∗∗ indicates 10%, 5% and 1% of significance probability level.

The coefficient of Mills ratio (Lamda) in the Heckman two-stage estimation was significant at the probability of less than 5%. This indicates sample selection bias and the existence of some unobservable household characteristics determining livelihood to participate in *enset* market. As per prior expectation, this variable influences positively and significantly marketed surplus at a 1% significance level. This indicates that as farmers’ years of schooling increase by one year, the intensity of participation increases in terms of marketed surplus by .121, ceteris paribus. This is because they produce in a more market-oriented manner than household heads with lower education levels. They are more capable of discovering pertinent information on enset production and marketing. The result is in line with the finding of Tessema et al. (2017); Geremewe et al. (2019); Mulatu (2021) who found that the educational level of households had a positive effect on the marketed surplus of *enset*.

Also, the amount of *enset* production affected the amount of marketed surplus positively and significantly at less than 1% level of significance. A one kg increase in the quantity produced for *enset* results marketed surplus of *enset* products by, 0.002 kg, ceteris paribus. This can be explained by the fact that the higher the produce the higher the farmers’ motivation to sell more to generate more income. This finding tallies with that of Kabeto (2014) who found that in Ethiopia when farmers produce redder beans, it motivates them to sell more. The higher the output, the higher is the farmer willing to participate in the market (Nuri and Jema, 2016; Mulatu, 2021). The findings of Adeoti et al. (2014); Gebreslassie (2015); Melaku et al. (2016); Mohammed et al. (2016); Geremewe et al. (2019) also affirm the importance of the size of production in determining the level of market participation.

Households having many years of *enset* producing experiences are more familiar with the benefit obtained from planting and cultivation activities and can easily know about the different input materials required for increasing the productivity of *enset*. This implies that ceteris paribus, an increase in years of farming experiences of household increases by one year, results from an increase in marketed surplus by 0.043 kg. This result is in line with the finding of Nuri and Jema (2016), who illustrated the positive relationship between experience and marketed surplus of *enset* product. The result is also in line with the finding of Shafi et al. (2014); Hailu (2016) showed a positive relationship between the experience of households in enset production and their market supply. Household size affected the amount of marketed surplus of *enset* products positively and significantly at a 1% level of significance. For a unit increase in household size (man equivalent), the marketed surplus from *enset* products increases by 0.1256 kg, ceteris paribus. Because production requires much more amount of labor, households have a large number of active labor forces more engaged in *enset* production and processing as well as marketing activities.

Livestock ownership influences the level of a marketed surplus of *enset* production in the study area. This variable affected the quantity of marketed surplus significantly and negatively at a 1% significance level. The result indicated that a unit increase in the number of livestock (TLU) owned by the households decreases the marketed surplus of *enset* by 0.262 quintals per year. The negative and significant coefficient of the variable depicts that, when households owed a large size of livestock herd, they would give much more time in the deployment of livestock and use *enset* as a supplementary feed. The finding is consistent with the finding of Bekele and Alemu (2015), Nuri and Jema (2016) and Mulatu (2021) who showed farmers with more TLU tend to specialize in livestock production reducing the importance of crop production as means of cash generation.

Non/off-farm income influences *enset* market participation decision significantly and negatively at less than 5% significance level. The model result confirms that a one birr increase in non/off-farm income of *enset* producer households leads to a decrease in marketed surplus by 3.8%. This may be explained by the fact that farmers who have better non/off-farm income would not tend to generate cash from sales (*enset* products) rather from their non/off-farm income. The result confirms the results of Nuri and Jema, 2016; Esmael et al. (2016) support this in explaining that income from non/off-farm has a negative relationship with enset market supply.

## 4 Conclusion and recommendation

*Enset* based agricultural production is one of the agricultural systems in Ethiopia which is commonly practiced in many parts of the densely populated south and south-western highlands of Ethiopia. Although *enset* is mainly cultivated as a staple food crop, it serves as a considerable income source for the growers. Factors that determine *enset* products market participation by farm households were analyzed by using the econometric model (Heckman selection model (two-step). Heckman's two-stage selection model showed that family size, level of education, farming experience, land allocation, livestock ownership, and access to training had significantly influenced market participation decision while family size, level of education, farming experience, livestock ownership, access to transport, quantity *enset* produced, off-farm income inverse Mill's ratio (LAMBDA) influenced significantly the extent of marketed surplus.

Based on the finding of the study the following policy implication is forwarded: introducing improved *enset* variety, encouraging the use of labor-saving technology, disseminating efficient processing devices, strengthening the existing extension package program, and promoting and empowering females. Thus, the government and/or private sector should encourage farmer training in the form of workshops regarding production, marketing, and value addition since it enables farmers to exchange ideas and experience on how to add more value to their *enset* products. Economical support should be given to farmers through formal credit agencies. Strong extension intervention is vital to assist farmers in producing high-quality enset products and increase production through consistent follow-up and keeping of farm records.

## Declarations

### Author contribution statement

Engida Gebre; Yaregal Tilahun; Benyam Tadesse; Kusse Haile; Tewdros Legesse: Analyzed and interpreted the data; Wrote the paper.

### Funding statement

This research did not receive any specific grant from funding agencies in the public, commercial, or not-for-profit sectors.

### Data availability statement

Data will be made available on request.

### Declaration of interests statement

The authors declare no conflict of interest.

### Additional information

No additional information is available for this paper.

## Figures and Tables

**Appendix Table 1:** Conversion factor for adult equivalent Age group (years) Adult equivalent

Age groups (years)		Adult equivalent
	Male	Female
11–13	0.9	0.8
14–16	1	0.75
17–50	1	0.75
>50	1	0.7

**Appendix Table 2:** conversion factor used to estimate tropical livestock unit equivalents.

No	Animal category	TLU1
1	Ox and caw	1.0
2	Sheep and goat (young)	1.06
3	Sheep and goat (adult)	0.13
4	Horse	1.1
5	Donkey (young)	0.35
6	Donkey (adult)	0.7
7	Heifer	0.75
8	Calf	0.25
9	Chickens	0.017

Source: stock et al.,(1991)

**Appendix Table 3:**
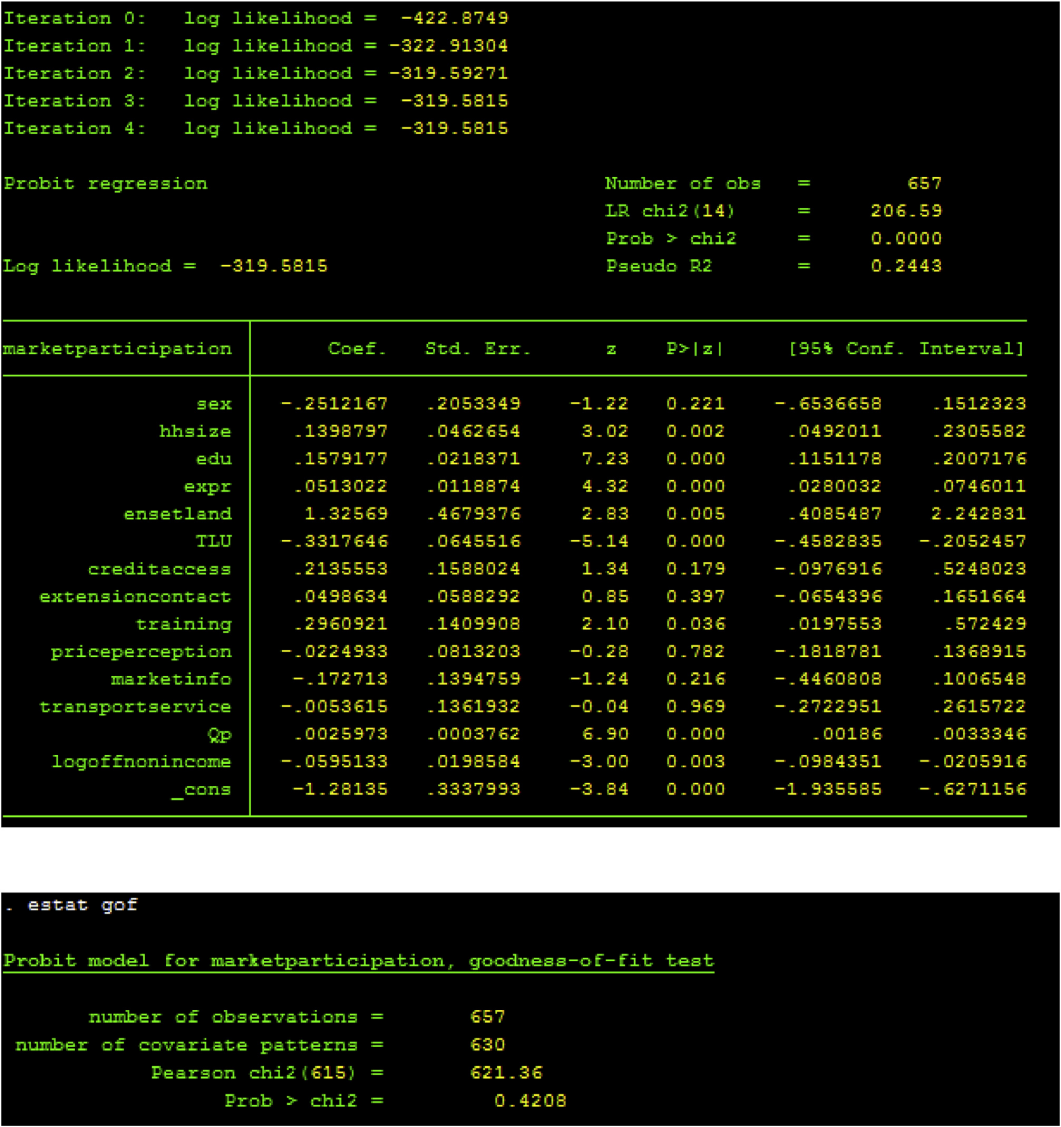
Probit regression result.

**Apeendix Table 4:**
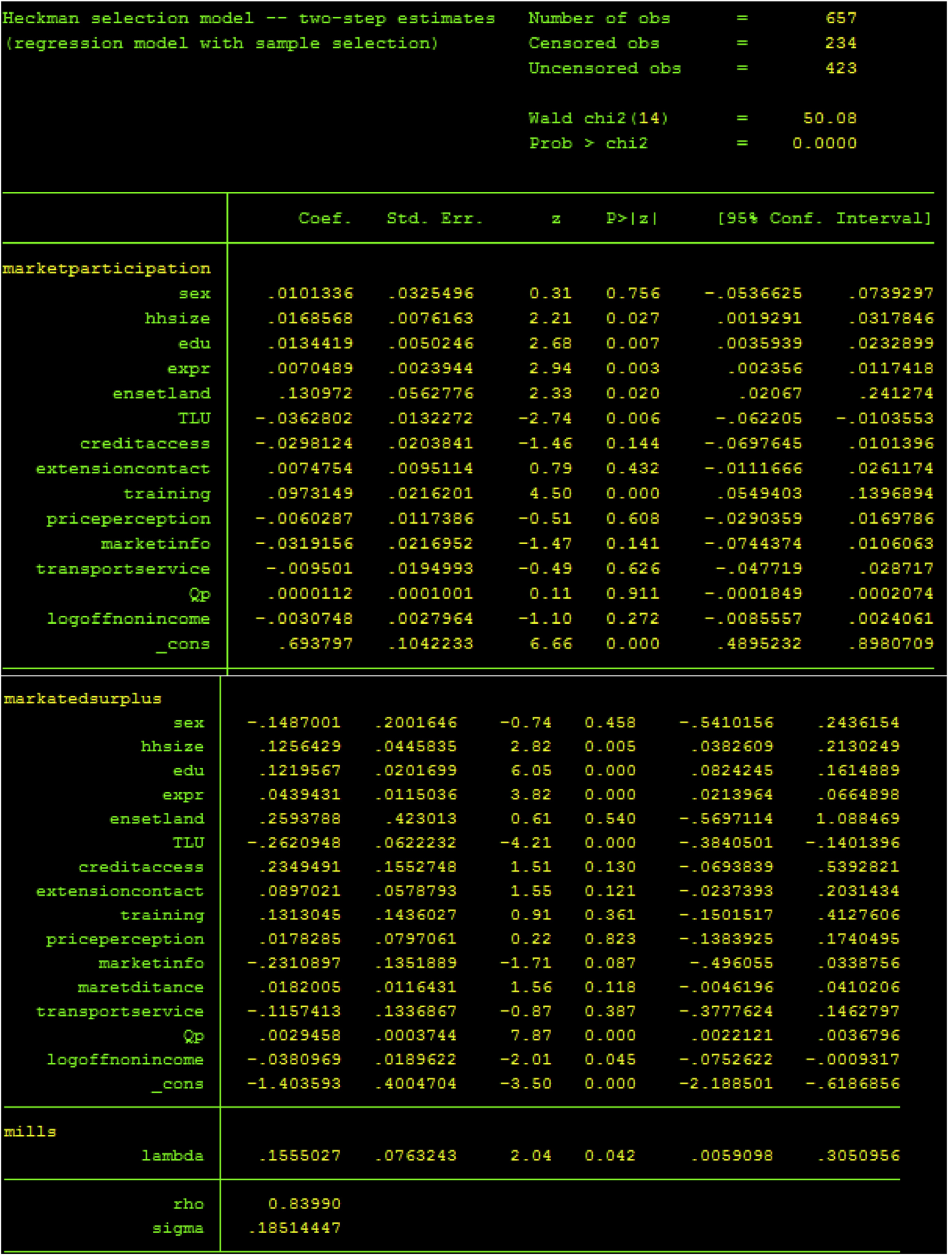
Heckman two-step regression result.
